# Enhanced removal of nickel(II) ions from aqueous solutions by SDS-functionalized graphene oxide

**DOI:** 10.1080/01496395.2016.1162172

**Published:** 2016-03-22

**Authors:** Elif Çalışkan Salihi, Jiabin Wang, Daniel J. L. Coleman, Lidija Šiller

**Affiliations:** ^a^ School of Chemical Engineering and Advanced Materials, Newcastle University, Newcastle upon Tyne, UK; ^b^ Faculty of Pharmacy, Department of Basic Pharmaceutical Sciences, Marmara University, Haydarpasa, Istanbul, Turkey; ^c^ Northern Institute for Cancer Research, Medical School, Newcastle University, Newcastle upon Tyne, UK

**Keywords:** Adsorption, graphene, nanomaterials

## Abstract

In this paper, a one-pot and easy-to-handle method at room temperature without additional chemicals for the modification of graphene oxide (GO) with surfactant is found. Removal of nickel (II) ions from aqueous solutions by GO and surfactant (sodium dodecyl sulphate) modified graphene oxide (SDS-GO) was studied spectrophotometrically at room temperature as a function of time, initial concentration and pH. Adsorption capacity of the adsorbent was increased dramatically (from 20.19 to 55.16 mg/g found by Langmuir model) due to the functionalization of the surface by SDS. The driving force of the adsorption of Ni(II) ions is electrostatic attraction and Ni(II) ions adsorbed on the GO surface chemically besides ion exchange.

## Introduction

Heavy metal pollution in the aquatic environment is a serious environmental problem. In recent years, several methods for the treatment of waste water contaminated with heavy metals have been extensively studied and adsorption is now recognized as an effective and economic approach. The adsorption process offers flexibility in design and operation of treatment processes as well as producing high-quality treated effluent in many cases.^[^ ^1^ ^–^ ^4^ ^]^ Adsorbents which have large surface area, pore volume and proper functionalities can be expected to perform most effectively and, for this reason, graphene oxide (GO) and graphene nanosheets have attracted tremendous interest. GO is functionalized graphene with various chemically bound oxygen-containing groups and is a potential adsorbent for metal (especially cationic metal) ion complexation through both electrostatic and coordination approaches due to reactive functional groups on GO surface.^[^ ^5^ ^]^

In the literature, there are a number of examples of the modification of GO with organics or metal oxides for the removal of metal ions from water.^[^ ^5^ ^–^ ^10^ ^]^ Madadrang et al. studied the modification of GO with ethylenediamine triacetic acid (EDTA) the resulting material displayed increased adsorption capacity for Pb (II) in comparison to GO.^[^ ^11^ ^]^ Ren et al. used a graphene/δ-MnO_2_ composite for the removal of Ni(II) ions from wastewater and obtained higher adsorption capacity with respect to graphene or MnO_2_ itself.^[^ ^12^ ^]^ Graphene/δ-MnO_2_ was prepared under 80–90°C which is an energy- and equipment-demanding method. Zawisza et al. used GO as a solid sorbent for the preconcentration of cobalt, nickel (Ni), copper, zinc and lead.^[^ ^13^ ^]^ The procedure in that study was based on dispersive micro-solid phase extraction and showed the great potential of GO as an excellent sorbent for preconcentration.^[^ ^13^ ^]^ Gaboardi et al. synthesized Ni-decorated graphene which showed increased hydrogen adsorption capacity compared to other common carbon-based materials.^[^ ^14^ ^]^ Ding et al. synthesized a reduced GO-supported chiral-modified Ni catalyst which they successfully employed for asymmetric hydrogenation.^[^ ^15^ ^]^

Ni is the 24th most abundant element in the Earth’s crust and is used in many industrial and commercial applications including electroplating, battery manufacture, forging, metal finishing and mining, all of which lead to environmental pollution by Ni. Exposure to highly Ni-polluted environments has the potential to produce various pathological effects in humans, such as contact dermatitis, lung fibrosis, cardiovascular and kidney diseases and cancer.^[^ ^16^ ^–^ ^19^ ^]^ Ni is also an excellent catalyst for carbon dioxide reforming of methane and methane autothermal reforming with CO_2_+O_2_ in a fluidized-bed reactor ^[20,21]^ for production of synthesis gas (CO and H_2_), which is the key step in the conversion of natural gas to liquid fuels and chemicals. Ferdowsi et al. reported Ni nanoparticle (NiNP)-modified graphite electrode for the electrocatalytic oxidation of methanol.^[22]^

The catalytic activity of Ni in the form of NiNPs for the reversible hydration of carbon dioxide at room temperature and atmospheric pressure has been recently reported.^[^ ^23^ ^]^ This behaviour is potentially important for CO_2_ capture technologies and for mineralization processes ^[23,24]^ and it has been confirmed that NiNPs are capable of accelerating mineral carbonation processes.^[23,25]^ Based on these results, technology to capture and mineralize CO_2_ in the presence of NiNPs has been proposed. In order for this technology to become widely acceptable, it is very important to develop robust Ni adsorbers as precaution against environmental accidents (*e.g*. after spillage dissolution of NiNPs into Ni(II) ions could occur under prolonged exposure to rainfall).

Therefore, there is an urgent need to develop a simple synthetic route and safe adsorber for removal of Ni(II) ions from water. Here we report an investigation into the use of GO for the removal of Ni(II) ions from aqueous solutions as a function of time, solute concentration, pH and adsorbent concentration. In addition, a one-pot and easy-to-handle method at room temperature without additional chemicals for the modification of GO surface using sodium dodecyl sulphate (SDS) was developed as it is expected to enhance Ni(II) ions removal based on previous study on the removal of drugs using bentonite in the presence of surface active agents by Çalışkan and Mahramanlıoğlu.^[^ ^26^ ^]^

## Materials and experimental method

### Materials

Natural graphite flakes (99.8% purity) were purchased from VWR (UK). Sulphuric acid (98%), phosphoric acid (85%), hydrogen peroxide (35%), potassium permanganate (99%), Ni(II) chloride and SDS were purchased from Sigma Aldrich (UK). All reagents were used without further purification. Deionized (DI) water produced by a Nanopure (Thermo Scientific, USA) purification system was used in all the experiments.

### Preparation and modification of **GO**

GO was produced by the oxidation and exfoliation of graphite with strong oxidants by the Hummers method,^[27]^ revised in our laboratory. Briefly, phosphoric acid was slowly added to sulphuric acid at room temperature under continuous mixing. Graphite was added to the solution and formed a homogeneous black dispersion. Potassium permanganate was then added into the solution slowly to avoid a sudden temperature change and caused the solution to become a dark green dispersion. This solution was left at room temperature for 3 days to achieve a complete reaction. Hydrogen peroxide was then dropped into the beaker until the solution became bright yellow in order to terminate the reaction. The resulting solution was first washed with 5% hydrochloric acid (HCl) and then washed by DI water several times until the pH value reached 7. After neutralization, the solution was dried in an oven at a temperature of 343 K to obtain GO powder. Modified GO (GO-SDS) was then prepared by stirring GO with SDS solution at a concentration of 1.2 g/L for 24 h at room temperature followed by washing with DI water several times in order to remove excess SDS. Once washed, the GO-SDS was also dried in the oven at 343 K.

### Adsorption experiments

Adsorption experiments were conducted by stirring known amounts of GO or GO-SDS (ranging from 10 to 100 mg) with 25 mL of aqueous Ni(II) solutions (at concentrations ranging from 5 to 40 mg/L) for predetermined time intervals at room temperature (298 K). The samples were filtered (Millipore HAWG047 S6, pore size 0.45 micrometre filter, Sigma Aldrich, UK) and concentrations were measured spectrophotometrically. Experiments measuring the kinetics of adsorption and the effect of pH were conducted using the same method. pH adjustment of Ni(II) solutions was achieved by adding concentrated HCl or NaOH to the solutions. Control experiments, with no adsorbent added to the solutions, were performed for each series of measurements. Experiments were conducted in triplicate under identical conditions and results were found to be reproducible (with an experimental error of approximately 3%). In order to calculate the concentration retained in the adsorbent phase (*q*, mg/g), the following equation was used:

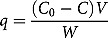

where *C*_0_ is the initial concentration of the adsorbate (mg/L) and *C* is the final concentration of the adsorbate (mg/L). *V* is the volume of the solution (L) and *W* is the mass of adsorbent (g) used in the experiments.

### Measurement and characterization

The concentrations of the Ni(II) solutions were measured by inductively coupled plasma optical emission spectroscopy (The ATI Unicam 701-Emission Inductively Coupled Argon Plasma Spectrophotometer, USA) and a UV-Visible spectrophotometer (Cary 100 UV-Vis, Agilent Technologies, USA) by applying the Dimethylglyoxime method.^[^ ^28^ ^]^ Fourier transform infrared (FTIR) spectra of the samples were recorded with a Varian 800 FTIR spectrometer. Scanning electron microscopy (SEM) measurements were performed on a Philips XL30 ESEM FEG environmental scanning electron microscope, Netherland. A zetasizer Nano ZS (Malvern, Instruments Ltd., UK) was used for zeta potential measurements using the setting for a polystyrene standard. Size of the samples was estimated by TEM from Philips CM-100, Netherlands with tungsten filament.

## Results

### Adsorption kinetics

**Figure 1.**
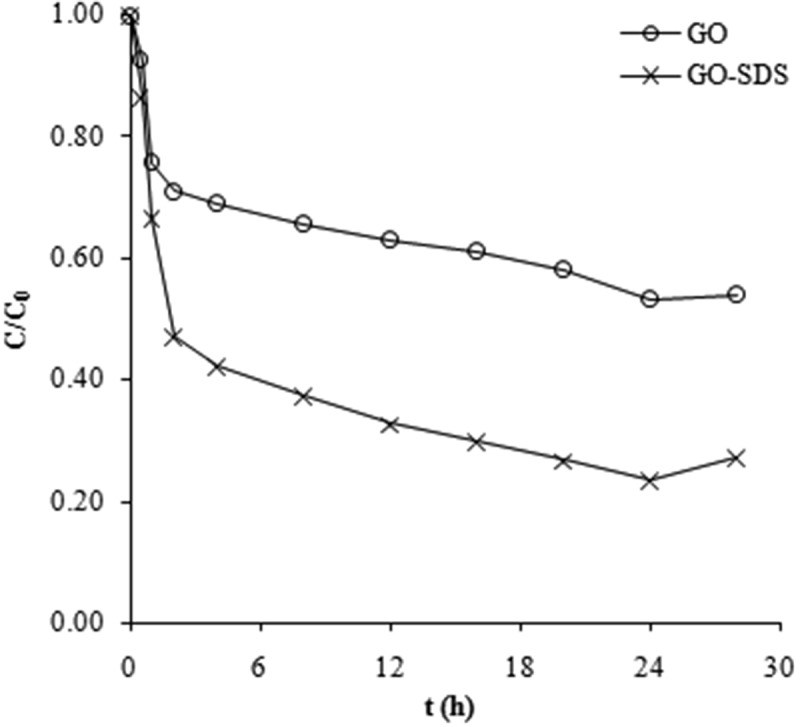
Effect of contact time on the adsorption of Ni(II) ions on graphene oxide (GO) and SDS-modified graphene oxide (GO-SDS) in aqueous solutions, denoted by open circle and crosses, respectively (the initial concentration of Ni(II) ions was 40 mg/L). The lines are a guide to the eye.

The effect of contact time on the adsorption of Ni(II) ions studied at an initial concentration of 40 mg/L is shown in Fig. 1. The concentration of Ni(II) ions solutions decreased with time due to adsorption of Ni(II) ions by the adsorbents, and the time to reach equilibrium was observed to be 24 h.

Adsorption rate constants for Ni(II) ions were calculated (Table 1) using Lagergren first-order and pseudo-second-order rate equations (Eqs. (1) and(2))^[^ ^29^ ^]^:

(1) 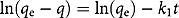

(2) 
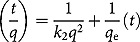

**Table 1.** Kinetic parameters for the adsorption of Ni(II) ions on graphene oxide (GO) and SDS-modified graphene oxide (GO-SDS).

Lagergren first-order model				Pseudo-second-order model			Intraparticle diffusion	
	*k*_1_ (1/h)	*q*_1_ (mg/g)	*R*_2_	*k*^2^ (g/mg h)	*q*_2_ (mg/g)	*R*_2_	*k*_d_ (mg/g h^1/2^)	*R*_2_
GO	0.1214	6.71	0.862	0.0597	18.08	0.998	0.99	0.977
GO-SDS	0.1425	18.00	0.942	0.0192	32.05	0.997	2.60	0.999

where *q* and *q*_e_ (both in mg/g) are the amounts of Ni(II) ions adsorbed at a time *t*, and at equilibrium respectively, *k*_1_ is the adsorption rate constant of the Lagergren first-order model and *k*_2_ is the adsorption rate constant of the pseudo-second-order model. Adsorption of Ni(II) ions was fitted to the pseudo-second-order model (Fig. 2), which shows better agreement with experimental data than the Lagergren first-order model (plot not shown), decided on the basis of the (*R* ^2^) correlation coefficient values.

**Figure 2.**
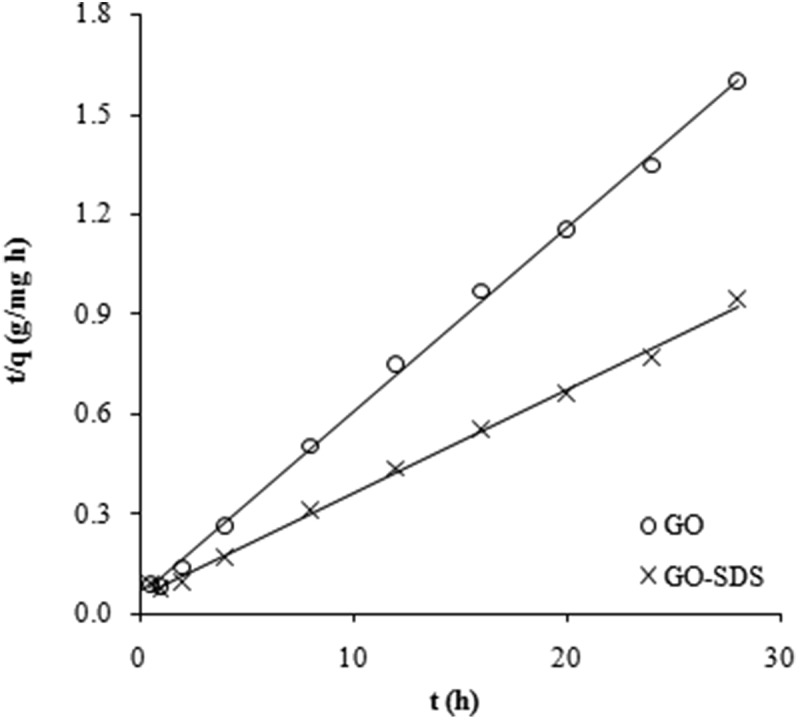
Adsorption of Ni(II) ions by GO (circles) and GO-SDS (crosses) fitted to the pseudo-second-order model (see text for details).

It is necessary to determine the rate-limiting step of the adsorption mechanism which has three steps. The rate controlling mechanism can be one or any combination of following:

1. Mass transfer across the external boundary layer film of the liquid surrounding the outside of the adsorbent.
2. Diffusion of the adsorbate molecules to an adsorption site either by a pore diffusion process through liquid filled pores or by a solid surface diffusion mechanism.
3. Adsorption at a site on the surface (internal or external), the energy of which will depend on the binding process (physical or chemical); this step is assumed to be extremely rapid.^[^ ^30^ ^]^

**Figure 3.**
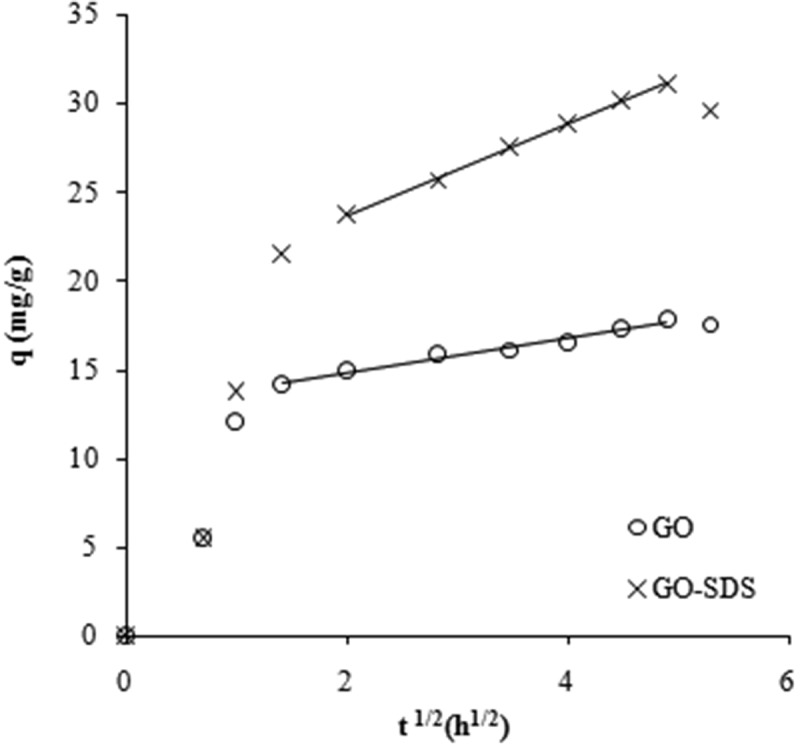
Intraparticle diffusion plots for graphene oxide (GO) and SDS-modified graphene oxide (GO-SDS) (denoted by open circles and crosses, respectively).

Intraparticle diffusion plots (Fig. 3) were used to analyse the mechanism of the adsorption in order to determine the rate-limiting step. To show the effect of intraparticle diffusion (Fig. 3) in the adsorption process, the amount of Ni(II) ions adsorbed at any time (*q*) was plotted against the square root of time (*t* ^1/2^).^[31,32]^ There is an initial steep curve followed by a straight line, which indicates that two mechanisms are operating in the removal of Ni(II) ions with a plateau that indicates the equilibrium region. The initial curve can be explained by the boundary layer effect while the linear part corresponds to intraparticle diffusion. The linear portions of the curves do not pass through the origin, denoting that intraparticle diffusion is not the only rate-controlling step for the adsorption of Ni(II) ions in this system.^[33]^ The rate constants of intraparticle diffusion were obtained from the slopes of the straight lines of the second parts of the plots and are presented in Table 1.

*k*_d_ is the intraparticle diffusion rate constant, *R* ^2^ is the correlation coefficient.

### Equilibrium isotherm models

**Figure 4.**
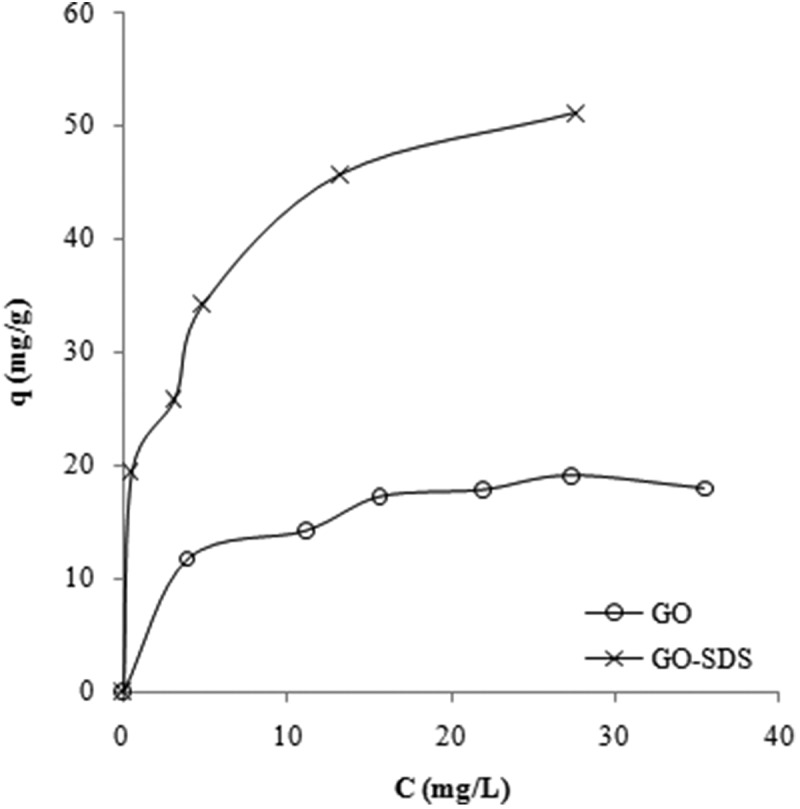
Giles isotherms of the adsorption of Ni(II) ions on graphene oxide (GO) and SDS-modified graphene oxide (GO-SDS),^[34]^ denoted by open circles and crosses, respectively. The lines are a guide to the eye.

According to the Giles isotherm classification, the shapes of the Giles isotherms for GO and GO-SDS (Fig. 4) shows ‘L type (subgroup 2)’ and ‘H-type (subgroup 2)’ behaviour, respectively. L-type behaviour is characteristic of systems where the adsorbate presents high affinity towards the adsorbent, and therefore indicates that no strong competition of the solvent with adsorbate takes place for the active sites of adsorption. H type is a special case of the L curve, in which the adsorbate has such high affinity to the adsorbent.^[34,^ ^35^ ^]^

Equilibrium data were analysed by using Langmuir ^[^ ^36^ ^]^ and Freundlich ^[^ ^37^ ^]^ isotherm models. Linear forms of the Langmuir and Freundlich isotherm equations are presented in Eqs. (3) and (4), respectively:

(3) 
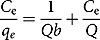

(4) 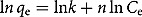

where *C_e_* is the final concentration at equilibrium in mg/L, *q_e_* is the amount of adsorbate adsorbed per unit mass of adsorbent at equilibrium in mg/g, *Q* is the maximum adsorption at monolayer coverage in mg/L and *b* is the adsorption equilibrium constant related to the energy of adsorption in L/mg. *k* and *n* are the Freundlich constants representing the adsorption capacity and intensity, respectively. The constants associated with the equations were determined and are shown in Table 2.

**Table 2.** Adsorption isotherm parameters for the adsorption of Ni(II) ions on graphene oxide (GO) and SDS-modified graphene oxide (GO-SDS).^[36,^ ^37^ ^]^

		Langmuir model			Freundlich model	
	*Q* (mg/g)	*b* (L/g)	*R*^2^	*n*	*k*	*R*^2^
GO	20.19	0.32	0.990	0.22	8.76	0.911
GO-SDS	55.16	0-40	0.992	0.26	22.03	0.960

*Q, b* = Langmuir constants; *n, k*= Freundlich constants.

**Figure 5.**
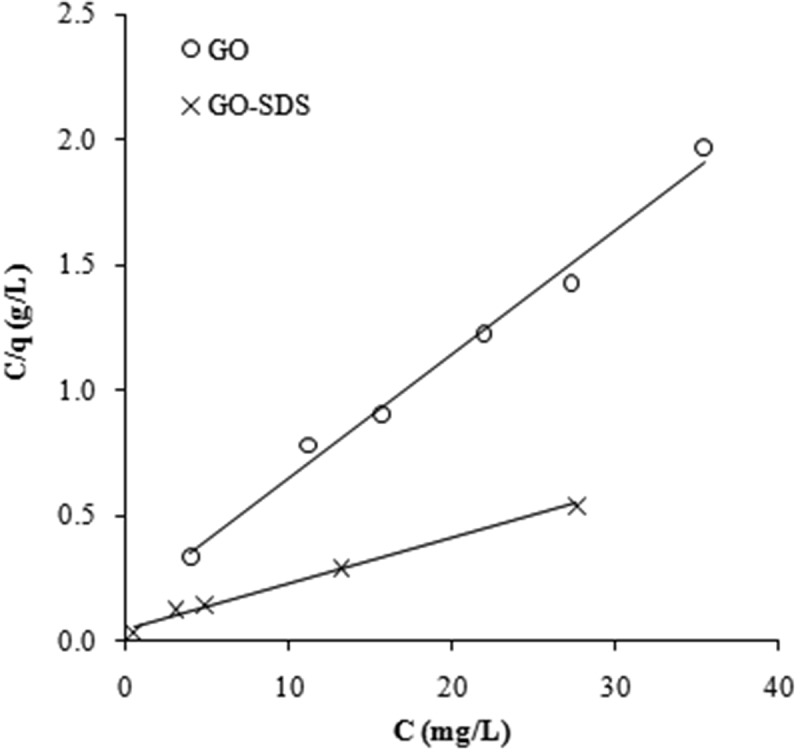
Langmuir isotherms fitted to the adsorption of Ni(II) ions on graphene oxide (GO) and SDS-modified graphene oxide (GO-SDS), denoted by open circles and crosses, respectively. See text for details.

The Langmuir model (Fig. 5) gave a better fit than the Freundlich model (plot not shown) for this process on the basis of the correlation coefficient (*R* ^2^) values (Table 2).

### Effect of pH

The pH of the solution is one of the most important factors affecting the adsorption of metal ions. This is partly because hydrogen ions themselves are strongly competing with metal ions. In all solutions, there has been competitive adsorption among hydronium ions (H_3_O^+^) and metal ions. At low pH values, hydronium ions are adsorbed more than other ions since hydronium ions have high concentration and more tendencies to be adsorbed. With increasing the pH, hydronium ion concentration is reduced and results in other ions being better and more adsorbed.^[38,^ ^39^ ^]^

**Figure 6.**
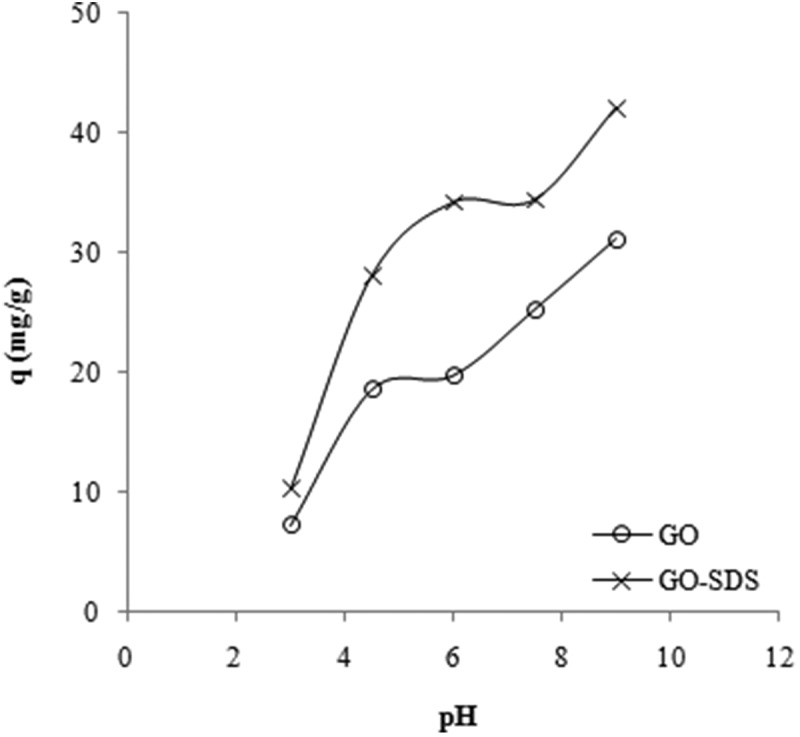
Effect of pH on the adsorption capacity (*q*) for the adsorption of Ni(II) ions on graphene oxide (GO) and SDS-modified graphene oxide (GO-SDS), denoted by open circles and crosses, respectively. The lines are a guide to the eye.

Ni can be present in the form of Ni(II) ions (*i.e*. Ni^2+^, Ni(OH)^+^, Ni(OH)_2_°, Ni(OH)_3_^–^ and Ni(OH)_4_^2^) in the medium depending on the pH of the solution.^[^ ^40^ ^]^ The effect of solution pH on the adsorption was studied at several pH values (between pH 3 and 9, where is the dominant species for Ni is the Ni^2+^ cation). Adsorption experiments were performed using an initial Ni(II) ions concentration of 40 mg/L at room temperature, and the results shown in Fig. 6. Solution pH had an important effect on the adsorption capacities for both of the adsorbents used (GO and GO-SDS) and both were significantly increased with increasing pH.

### Characterization and adsorption mechanism of Ni(II) ions

**Figure 7.**
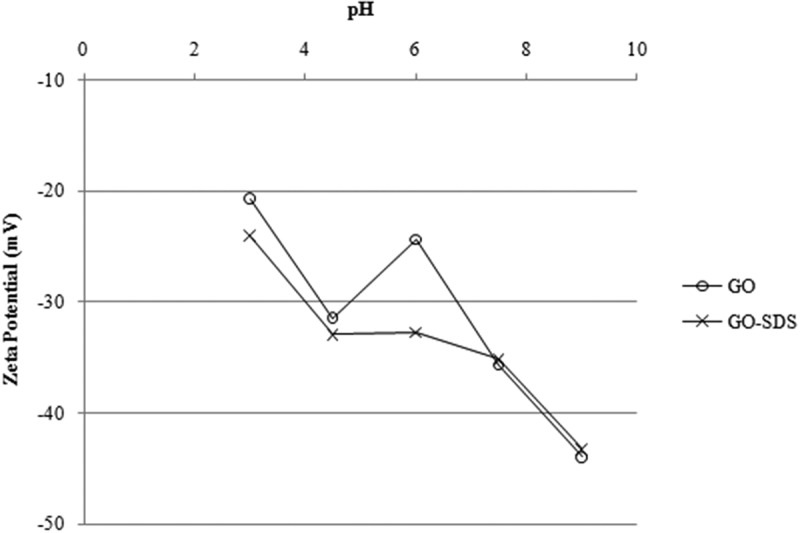
Change of zeta potential of graphene oxide (GO) and SDS-modified graphene oxide (GO-SDS) with pH, denoted by open circles and crosses, respectively.

Ni(II) ions adsorption on a carbon adsorbent may occur due to several mechanisms such as physical adsorption, chemical adsorption, ion exchange or a combination of these. In order to further understand the adsorption mechanism of Ni(II) ions, zeta potentials of the adsorbents at several pH values (between 3 and 9) were measured and are shown in Fig. 7. As seen in Fig. 7, zeta potentials of GO and GO-SDS are negative at all pH values studied, which confirms that the surface charge is negative ^[^ ^41^ ^]^, and mainly decreases from pH 3 to 9. The negative zeta potential even in acidic conditions indicates that GO and GO-SDS forms stable colloids due to electrostatic repulsion of the ionized functional groups.^[42]^

Zeta potential is a physical parameter used to quantify the adsorbent surface charge. By measuring the zeta potential as a function of pH, the acidity or basicity of the adsorbent surfaces can be determined. The zeta potentials of GO-SDS were more negative than those of GO and were all negative values within the pH range tested herein. This is probably due to the presence of negative functional groups introduced by modification. Numerous investigations have also demonstrated that the zeta potentials of modified adsorbents are more negative than those of as-produced ones, and it depends on the type of treatment for modification.^[43–^ ^46^ ^]^

The trend of the zeta potential is in accord with the increase in adsorption capacities at higher pH values. The effect of pH on adsorption and on zeta potentials of the adsorbents shows that the driving force of the Ni(II) ions adsorption on GO/GO-SDS surface is electrostatic attraction between a negatively charged adsorbent surface and positively charged Ni(II) ions. It is also observed that the surface of GO-SDS is more negative than the surface of GO which explains the higher adsorption capacity found for GO-SDS.

**Figure 8.**
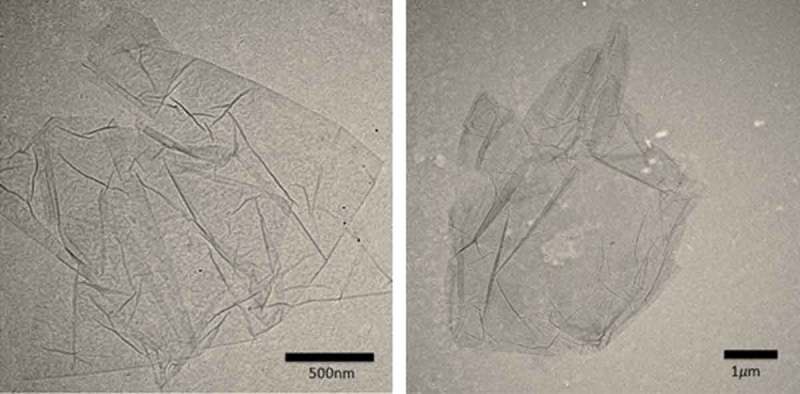
TEM images of graphene oxide (GO).

GO has a thin-layer structure, so dynamic light scattering (DLS) could not be used for particle size analysis to measure the size of GO and GO-SDS. DLS technique does not give accurate results for non-spherical particles. However, transmission electron microscope (TEM) images (Fig. 8) were used to estimate the size of GO. From these figures, GO has size larger than 2 µm. SDS works as functional groups on GO, so it can be assumed that GO-SDS has similar size with GO.

**Figure 9.**
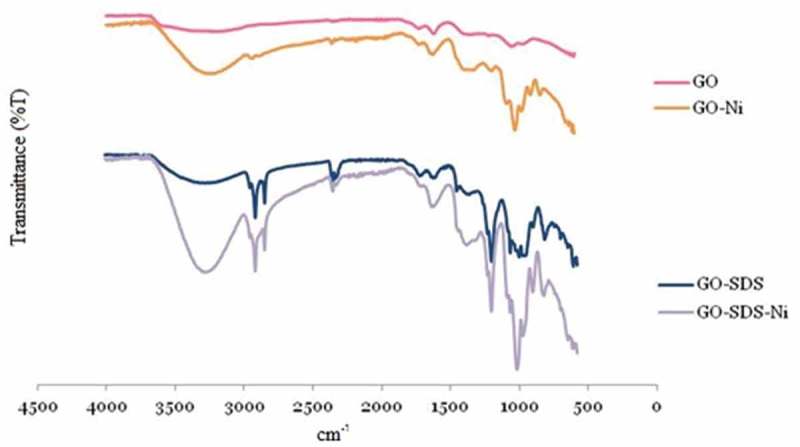
FTIR spectra of graphene oxide (GO) and SDS-modified graphene oxide (GO-SDS) before and after the adsorption of Ni(II) ions.

Figure 9 shows FTIR spectra obtained from GO and GO-SDS before and after the uptake of Ni(II) ions. The broad absorption band at between 3000 and 3500 cm^–1^ can be attributed to OH groups. Absorption bands at around 1650 cm^–1^ are due to carbonyl and carboxyl groups. Bands at around 1400 and 950 cm^–1^ are due to C–O bonds of hydroxyl or epoxy groups.^[^ ^47^ ^–^ ^49^ ^]^ Sharp peaks at around 2800 cm^–1^ appeared in the spectra of GO-SDS (after the treatment of GO with SDS) which are associated with C-H stretching. A sharp peak at around 1200 cm^–1^ also appeared in the spectra of GO-SDS which is due to the sulphate groups of SDS located on GO. The peaks at around 950 cm^–1^ became sharp and changed shape, which could be attributed to sulphate and hydroxyl groups from SDS.^[50]^ After contacting with Ni, a new sharp peak at 1020 cm^–1^ was observed for both of the adsorbents due to Ni(II) ions adsorption.^[^ ^51^ ^]^ According to the FTIR spectra, SDS does not interact with the GO surface in a covalent manner when GO-SDS is formed.

Physicochemical modification methods had been most widely adopted, namely covalent surface modification and non-covalent surface modification, depending on whether or not covalent bonding between the carbon structure and the functional groups and/or modifier molecules is involved in the surface modification process. The advantage of non-covalent functionalization is that it does not destroy the conjugation in the carbon structure. Non-covalent functionalization strategies do not have any effect on the physical properties of the adsorbent because they keep the structure of intrinsic *sp* ^2^ hybridized orbital unchanged. This can be done by taking advantage of the π interaction between conjugated molecules and the graphitic structure.^[52–^ ^54^ ^]^

**Figure 10.**
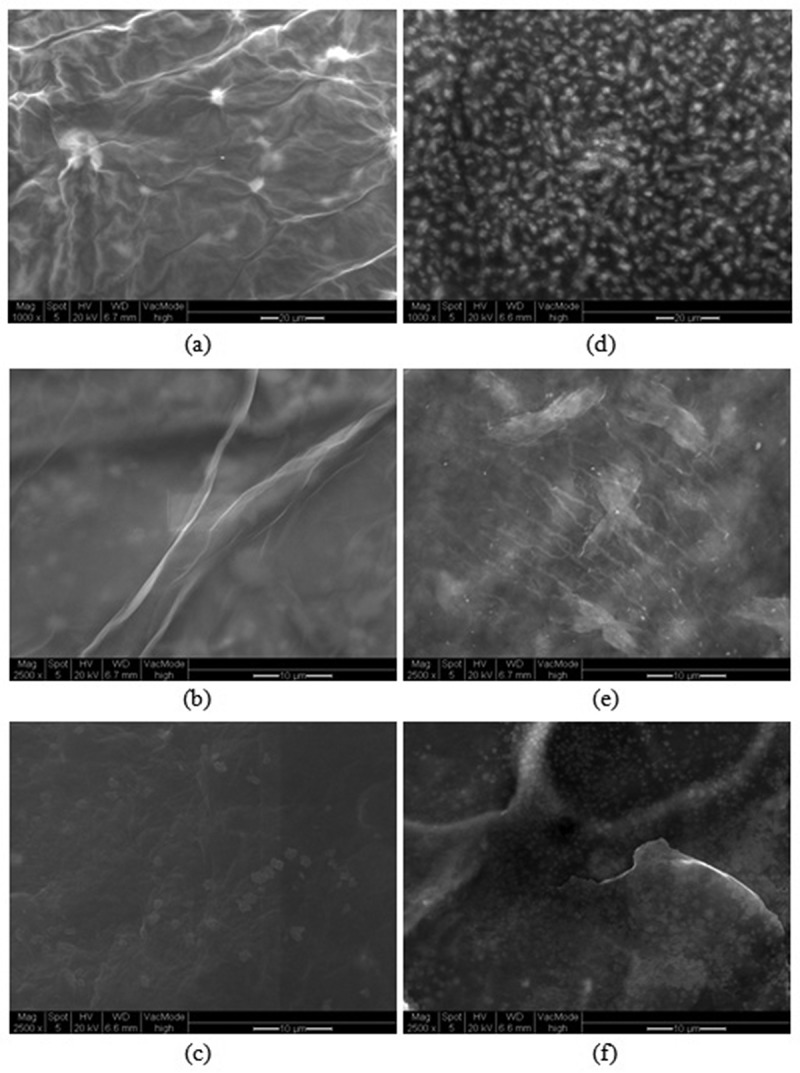
SEM images of graphene oxide (GO) and SDS-modified graphene oxide (GO-SDS) before and after the adsorption of Ni(II) ions. (a, b) GO; (c) GO-Ni; (d, e) GO-SDS; (f) GO-SDS-Ni. The scale bar in (a) and (d) is 20 µm and in (b), (c), (e) and (f) is 10 µm.

SEM images of GO and GO-SDS before and after Ni(II) ions adsorption are shown in Fig. 10. The surfactant layer on GO which forms GO-SDS can be seen in Fig. 10d, e (as globules and strands, respectively). As seen in Fig. 9, chemisorption can occur by means of surface complexation of Ni(II) ions with carbonyl and/or carboxyl groups of the adsorbent surface. Ni(II) ions reduced the adsorbent surface and formed NiO with flower-like shape on the surface (Fig. 10c, f). Another possible mechanism for the uptake of Ni(II) is ion exchange (cation exchange) of Ni(II) cations with hydrogens of hydroxyl and/or carboxyl groups of the adsorbent surface.

**Figure 11.**
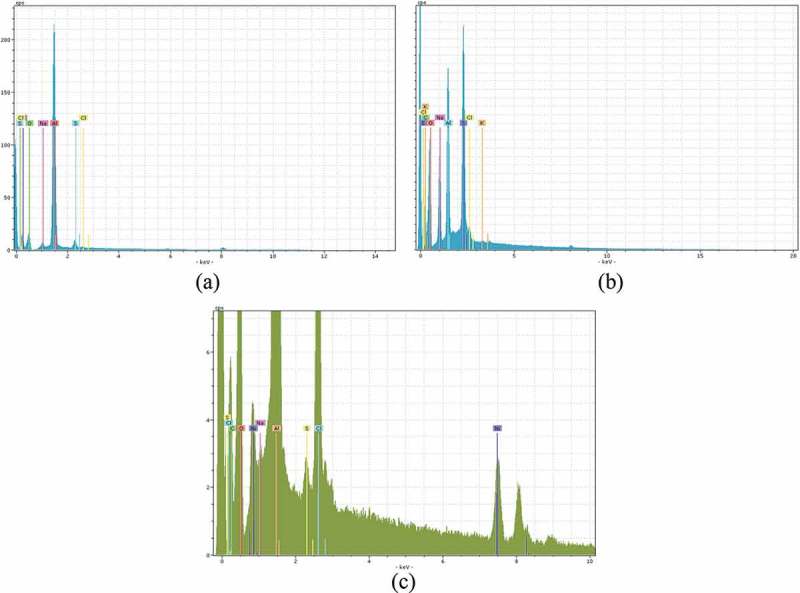
EDX results for graphene oxide (GO) (a); SDS-modified graphene oxide (GO-SDS) before (b) and after (c) the adsorption of Ni(II) ions.

Figure 11 shows the energy-dispersive X-ray spectroscopy (EDX) spectrum of GO and GO-SDS before and after the adsorption of Ni(II) ions. In the EDX spectrum of GO-SDS after the adsorption of Ni(II) ions (Fig. 11c), two new Ni(II) peaks emerged. This result confirmed the presence of Ni(II) ions on the GO-SDS surface.

**Figure 12.**
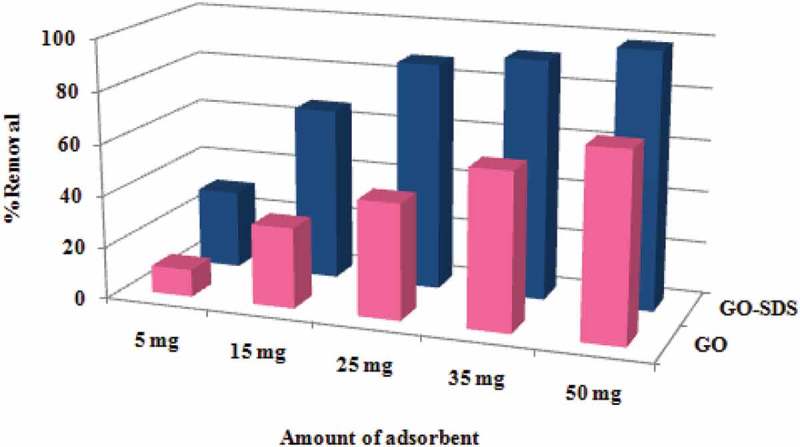
Comparison of % removal for different amounts of GO and GO-SDS

Figure 12 shows a brief comparison of removal of Ni(II) ions with GO and GO-SDS at different amounts. The modification of GO with SDS dramatically increases the uptake of Ni(II) ions, although it does not change the time to reach equilibrium.

## Conclusions

Adsorption technology is currently being applied extensively to the removal of organic and inorganic micropollutants from aqueous solutions, and carbon nanostructures with different morphologies are assumed to be one of the major elements in nanotechnology.^[55,56]^ GO produced is an effective adsorbent for the removal of Ni(II) ions from aqueous solutions and has the potential to be used for the treatment of waste or drinking water. In the literature, there are examples of the modification of GO with organics or metal oxides ^[^ ^10^ ^–^ ^15^ ^]^ for the removal of metal ions from water. Ren et al. have used graphene/ δ-MnO_2_ for the removal of Ni (II) from aqueous solutions and found the adsorption capacity as 46.55 mg/g ^[^ ^12^ ^]^ which is lower than that we found in our study (55.16 mg/g). Graphene/δ-MnO_2_ was prepared under 80–90°C which is an energy- and equipment-intensive method. Zhang and Wang^[57]^ have studied the adsorption of Ni(II) ions between the temperatures of 40°C and 75°C. The adsorption capacity at 25°C was not reported. Additionally, we can understand from Zhang and Wang’s study that the adsorption capacity of Ni(II) ions on lignocellulose/montmorillonite nanocomposite tends to increase with temperature. They have found the adsorption capacity around 67 mg/g at 40°C and around 95 mg/g at 70°C. We have studied the adsorption of Ni(II) ions at room temperature (25°C), so Zhang and Wang’s study is not comparable to our study. Table 3 shows a comparison of various adsorbents from literature data for the adsorption of Ni(II) ions that are comparable to our study.

**Table 3.** Comparison of various adsorbents for the removal of Ni^2+^.

Adsorbent	Adsorbate	mg/g (25°C)	Reference
Na-montmorillonite	Ni	3.63	^[^^58^^]^
Multiwalled carbon nanotubes	Ni	6.89	^[^^59^^]^
γ-Fe_2_O_3_	Ni	23.60	^[^^60^^]^
EDTA-chitosan	Ni	24.35	^[^^61^^]^
DTPA*-chitosan	Ni	24.16	^[^^61^^]^
Vermiculite	Ni	25.33	^[^^58^^]^
Nano-alumina	Ni	30.82	^[^^62^^]^
Activated carbon	Ni	34.72	^[^^63^^]^
GO-G	Ni	36.63	^[^^64^^]^
Graphene/δ-MnO_2_	Ni	46.55	^[^^12^^]^
GO-SDS	Ni	55.16	This study

*Diethylenetriaminepentaacetic acid

In this paper, a one-pot and easy-to-handle method at room temperature without additional chemicals for the modification of GO with surfactant is developed. Removal of Ni(II) ions from aqueous solutions by GO and SDS-GO was studied spectrophotometrically at room temperature as a function of time, initial concentration and pH. Adsorption capacity of the adsorbent was increased dramatically (from 20.19 mg/g to 55.16 mg/g found by Langmuir model) due to the functionalization of the surface by SDS. In this work, we show that the removal of Ni(II) ions from aqueous solutions onto GO/GO-SDS is highly sensitive to pH changes. The driving force of the adsorption of Ni(II) ions is electrostatic attraction and Ni(II) ions adsorbed on the GO surface chemically besides ion exchange.

## References

[CIT0001] Nagajyoti P.C., Lee K.D., Sreekanth T.V.M. (2010). Heavy metals, occurrence and toxicity for plants: a review. *Environmental Chemistry Letters*.

[CIT0002] Demirbas A. (2008). Heavy metal adsorption onto agro-based waste materials: a review. *Journal of Hazardous Materials*.

[CIT0003] Fu F., Wang Q. (2011). Removal of heavy metal ions from wastewaters: a review. Journal of Environmental Management.

[CIT0004] Hua M., Zhang S., Pan B., Zhang W., Lv L., Zhang Q. (2012). Heavy metal removal from water/wastewater by nanosized metal oxides: a review. *Journal of Hazardous Materials*.

[CIT0005] Wang S., Sun H., Ang H.M., Tadé M.O. (2013). Adsorptive remediation of environmental pollutants using novel graphene-based nanomaterials. *Chemical Engineering Journal*.

[CIT0006] He Y.Q., Zhang N.N., Wang X.D. (2011). Adsorption of graphene oxide/chitosan porous materials for metal ions. *Chinese Chemical Letters*.

[CIT0007] Chowdhury S., Balasubramanian R. (2014). Recent advances in the use of graphene-family nanoadsorbents for removal of toxic pollutants from wastewater. *Advances in Colloid and Interface Science*.

[CIT0008] Lee Y.-C., Yang J.-W. (2012). Self-assembled flower-like TiO_2_ on exfoliated graphite oxide for heavy metal removal. *Journal of Industrial and Engineering Chemistry*.

[CIT0009] Liu M., Chen C., Hu J., Wu X., Wang X. (2011). Synthesis of Magnetite/Graphene Oxide Composite and Application for Cobalt (II) Removal. *The Journal of Physical Chemistry C*.

[CIT0010] Zhang N., Qiu H., Si Y., Wang W., Gao J. (2011). Fabrication of highly porous biodegradable monoliths strengthened by graphene oxide and their adsorption of metal ions. *Carbon*.

[CIT0011] Madadrang C.J., Kim H.Y., Gao G., Wang N., Zhu J., Feng H., Gorring M., Kasner M.L., Hou S. (2012). Adsorption behavior of EDTA-graphene oxide for Pb (II) removal. *ACS Applied Materials & Interfaces*.

[CIT0012] Ren Y., Yan N., Wen Q., Fan Z., Wei T., Zhang M., Ma J. (2011). Graphene/δ-MnO_2_ composite as adsorbent for the removal of nickel ions from wastewater. *Chemical Engineering Journal*.

[CIT0013] Zawisza B., Sitko R., Malicka E., Talik E. (2013). Graphene oxide as a solid sorbent for the preconcentration of cobalt, nickel, copper, zinc and lead prior to determination by energy-dispersive X-ray fluorescence spectrometry. *Analytical Methods*.

[CIT0014] Gaboardi M., Bliersbach A., Bertoni G., Aramini M., Vlahopoulou G., Pontiroli D., Mauron P., Magnani G., Salviati G., Züttel A., Ricco M. (2014). Decoration of graphene with nickel nanoparticles: study of the interaction with hydrogen. *Journal of Materials Chemistry A*.

[CIT0015] Ding C., Wei W., Sun H., Ding J., Ren J., Qu X. (2014). Reduced graphene oxide supported chiral Ni particles as magnetically reusable and enantioselective catalyst for asymmetric hydrogenation. *Carbon*.

[CIT0016] Coman V., Robotin B., Ilea P. (2013). Nickel recovery/removal from industrial wastes: A review. *Resources, Conservation and Recycling*.

[CIT0017] Huang L., Sun Y., Yang T., Li L. (2011). Adsorption behavior of Ni(II) on lotus stalks derived active carbon by phosphoric acid activation. *Desalination*.

[CIT0018] Gupta V.K., Jain C.K., Ali I., Sharma M., Saini V.K. (2003). Removal of cadmium and nickel from wastewater using bagasse fly ash—a sugar industry waste. *Water Research*.

[CIT0019] Villaescusa I., Fiol N., Martinez M., Miralles N., Poch J., Serarols J. (2004). Removal of copper and nickel ions from aqueous solutions by grape stalks wastes. *Water Research*.

[CIT0020] He S., Jing Q., Yu W., Mo L., Lou H., Zheng X. (2009). Combination of CO_2_ reforming and partial oxidation of methane to produce syngas over Ni/SiO_2_ prepared with nickel citrate precursor. *Catalysis Today*.

[CIT0021] Guo J., Gao J., Chen B., Hou Z., Fei J., Lou H., Zheng X. (2009). Catalytic conversion of CH_4_ and CO_2_ to synthesis gas on Ni/SiO_2_ catalysts containing Gd_2_O_3_ promoter. *International Journal of Hydrogen Energy*.

[CIT0022] Gh Ferdowsi, Seyedsadjadi S., Ghaffarinejad, A. S. A. (2015). Ni nanoparticle modified graphite electrode for methanol electrocatalytic oxidation in alkaline media. *Journal of Materials Chemistry A*.

[CIT0023] Bhaduri G.A., Šiller L. (2013). Nickel nanoparticles catalyse reversible hydration of carbon dioxide for mineralization carbon capture and storage. *Catalysis Science & Technology*.

[CIT0024] Bhaduri G.A., Henderson R.A., Šiller L. (2013). Reply to the ‘Comment on “Nickel nanoparticles catalyse reversible hydration of carbon dioxide for mineralization carbon capture and storage”‘ by D. Britt. Catal. Sci. Technol., 2013, 3.

[CIT0025] Bodor M., Santos R.M., Chiang Y.W., Vlad M., Van Gerven T. (2014). Impacts of nickel nanoparticles on mineral carbonation. *Science World Journal*.

[CIT0026] Çalışkan Salihi E., Mahramanlıoğlu M. (2014). Equilibrium and kinetic adsorption of drugs on bentonite: Presence of surface active agents effect. *Applied Clay Science*.

[CIT0027] Hummers W.S., Offeman R.E. (1958). Preparation of Graphitic Oxide. *Journal of the American Chemical Society*.

[CIT0028] Ortiz N., Pires M.A.F., Bressiani J.C. (2001). Use of steel converter slag as nickel adsorber to wastewater treatment. *Waste management*.

[CIT0029] Lewinsky A.A. (2007). *Hazardous Materials and Wastewater: Treatment, Removal and Analysis;*.

[CIT0030] Cheung W.H., Szeto Y.S., McKay G. (2007). Intraparticle diffusion processes during acid dye adsorption onto chitosan. *Bioresource Technology*.

[CIT0031] Mahramanlıoğlu M., Kızılcıklı İ., Biçer İ. Ö. (2002). Adsorption of fluoride from aqueous solutionby acid treated spent bleaching earth. *Journal of Fluorine Chemistry*.

[CIT0032] Çalışkan E., Göktürk S. (2010). Adsorption Characteristics of Sulfamethoxazole and Metronidazole on Activated Carbon. *Separation Science and Technology*.

[CIT0033] Rida K., Bouraoui S., Hadnine S. (2013). Adsorption of methylene blue from aqueous solution by kaolin and zeolite. *Applied Clay Science*.

[CIT0034] Giles C.H., Macewan T.H., Nakhwa S.N., Smith D.J. (1960). Studies in adsorption. Part XI. A system of classification of solution adsorption isotherms, and its use in diagnosis of adsorption mechanisms and in measurement of specific surface areas of solids. *Journal of the Chemical Society*.

[CIT0035] Mestre A.S., Pires J., Nogueira J.M., Parra J.B., Carvalho A.P., Ania C.O. (2009). Waste-derived activated carbons for removal of ibuprofen from solution: role of surface chemistry and pore structure. *Bioresource Technology*.

[CIT0036] Langmuir I. (1918). The Adsorption of Gases on Plane Surfaces of Glass, Mica and Platinum. *Journal of the American Chemical Society*.

[CIT0037] Freundlich H.M.F. (1906). Über die adsorption in lösungen. *Zeitschrift für Physikalische Chemie*.

[CIT0038] Moradi O., Aghaie M., Zare K., Monajjemi M., Aghaie H. (2009). The Study of AdsorptionCharacteristics Cu2+ and Pb2+ Ions onto PHEMA and P(MMA-HEMA) Surfaces from Aqueous Single Solution. *Journal of Hazardous Materials*.

[CIT0039] Moradi O., Zare K., Monajjemi M., Yari M., Aghaie H. (2010). The Studies of equilibrium and thermodynamic adsorption of Pb(II), Cd(II) and Cu(II) Ions from Aqueous solution onto SWCNTs and SWCNT –COOH Surfaces, Fullerenes. *Nanotubes and Carbon Nanostructures*.

[CIT0040] Liu H., Wang X., Zhai G., Zhang J., Zhang C., Bao N., Cheng C. (2012). Preparation of activated carbon from lotus stalks with the mixture of phosphoric acid and pentaerythritol impregnation and its application for Ni(II) sorption. *Chemical Engineering Journal*.

[CIT0041] Zhang W., Zhou C., Zhou W., Lei A., Zhang Q., Wan Q., Zou B. (2011). Fast and considerable adsorption of methylene blue dye onto graphene oxide. *Bulletin of Environmental Contamination and Toxicology*.

[CIT0042] Ramesha G.K., Kumara A.V., Muralidhara H.B., Sampath S. (2011). Graphene and graphene oxide as effective adsorbents toward anionic and cationic dyes. *Journal of Colloid and Interface Science*.

[CIT0043] Wu C. (2007). Studies of the equilibrium and thermodynamics of the adsorption of Cu2+ onto as-produced and modified carbon nanotubes. *Journal of Colloid and Interface Science*.

[CIT0044] Li Y., Ding J., Luan Z., Di Z., Zhu Y., Xu C., Wu D., Wei B. (2003). Competitive adsorption of Pb, Cu and Cd ions from aqueous solutions by multiwalled carbon nanotubes. *Carbon*.

[CIT0045] Li Y., Wang S., Luan Z., Ding J., Xu C., Wu D. (2003). Adsorption of cadmium(II) from aqueous solution by surface oxidized carbon nanotubes. *Carbon*.

[CIT0046] Lu C., Chiu H. (2006). Adsorption of zinc(II) from water with purified carbon nanotubes. *Chemical Engineering Science*.

[CIT0047] Sun X., Liu Z., Welsher K., Robinson J.T., Goodwin A., Zaric S., Dai H. (2008). Nano-Graphene Oxide for Cellular Imaging and Drug Delivery. *Nano Research*.

[CIT0048] Wang G., Wang B., Park J., Yang J., Shen X., Yao J. (2009). Synthesis of enhanced hydrophilic and hydrophobic graphene oxide nanosheets by a solvothermal method. *Carbon*.

[CIT0049] Wang H., Hao Q., Yang X., Lu L., Wang X. (2009). Graphene oxide doped polyaniline for supercapacitors. *Electrochemistry Communications*.

[CIT0050] Eggleston C.M., Hug S., Stumm W., Sulzberger B., Dos Santos Afonso, M. (1998). Surface complexation of sulfate by hematite surfaces: FTIR and STM observations. *Geochimica et Cosmochimica Acta*.

[CIT0051] Panda G.C., Das S.K., Bandopadhyay T.S., Guha A.K. (2007). Adsorption of nickel on husk of Lathyrus sativus: behavior and binding mechanism. *Colloids and Surfaces B: Biointerfaces*.

[CIT0052] Moradi O., Yari M., Zare K., Mirza B., Najafi F. (2012). Carbon Nanotubes: Chemistry Principles and Reactions: Review. *Fullerenes, Nanotubes and Carbon Nanostructures*.

[CIT0053] Ahmadpour A., Eftekhari N., Ayati A. (2014). Performance of MWCNTs and a low-cost adsorbent for Chromium (VI) ion removal. *Journal of Materials Chemistry A*.

[CIT0054] Zare K., Gupta V. K., Moradi O., Makhlou A., Sillanpää S. H., Nadagouda, M. N. M., Sadegh H., Shahryari-ghoshekandi R., Pal A., Wang Z., Tyagi I., Kazemi M. (2015). A comparative study on the basis of adsorption capacity between CNTs and activated carbon as adsorbents for removal of noxious synthetic dyes: a review. *Journal of Nanostructure in Chemistry*.

[CIT0055] Parlayici S., Eskizeybek V., Avcı A., Pehlivan E. (2015). Removal of chromium (VI) using activated carbon-supportedfunctionalized carbon nanotubes. *Journal of Materials Chemistry A*.

[CIT0056] Anbia M., Khoshbooei S. (2015). Functionalized magnetic MCM-48 nanoporous silica by cyanuric chloride for removal of chlorophenol and bromophenol from aqueous media. *Journal of Nanostructure in Chemistry*.

[CIT0057] Zhang X., Wang X. (2015). Adsorption and Desorption of Nickel(II) Ions from Aqueous Solutionby a Lignocellulose/Montmorillonite Nanocomposite. *PLoS ONE*.

[CIT0058] Abollino O., Giacomino A., Malandrino M., Mentasti E. (2008). Interaction of metal ions with montmorillonite and vermiculite. *Applied Clay Science*.

[CIT0059] Liang P., Yan L., Guo L., Zeng J., Lu H. (2004). Multiwalled carbon nanotubes as solid-phase extraction adsorbent for the preconcentration of trace metal ions and theirdetermination by inductively coupled plasma atomic emission spectrometry. *Journal of Analytical Atomic Spectrometry*.

[CIT0060] Hu J., Chen G.H., Lo I.M.C. (2006). Selective removal of heavy metals from industrial waste water using maghemite nanoparticle: Performance and mechanisms. *Journal of Environmental Engineering (ASCE)*.

[CIT0061] Repo E., Warcholc J. K., Kurniawana T. A., Sillanpää M. E. T. (2010). Adsorption of Co(II) and Ni(II) by EDTA- and/or DTPA-modified chitosan: Kinetic and equilibrium modeling. *Chemical Engineering Journal*.

[CIT0062] Srivastava V., Weng C. H., Singh V. K., Sharma Y. C. (2011). Adsorption of Nickel Ions from Aqueous Solutions by Nano Alumina: Kinetic, Mass Transfer, and Equilibrium Studies. *Journal of Chemical & Engineering Data*,56.

[CIT0063] Guo Z., Fan J., Zhang J., Kang Y., Liu H., Jiang L., Zhang C. (2016). Sorption heavy metal ions by activated carbons with well-developed microporosity and amino groups derived from Phragmitesaustralis by ammoniumphosphates activation. *Journal of the Taiwan Institute of Chemical Engineers*.

[CIT0064] Najafi F., Moradi O., Rajabi M., Asif M., Tyagi I., Agarwal S., Gupta V.K. (2015). Thermodynamics of the adsorption of nickel ions from aqueous phaseusing graphene oxide and glycine functionalized graphene oxide. *Journal of Molecular Liquids*.

